# Silencing of circRNA circ_0001666 Represses EMT in Pancreatic Cancer Through Upregulating miR-1251 and Downregulating SOX4

**DOI:** 10.3389/fmolb.2021.684866

**Published:** 2021-05-13

**Authors:** Rundong Zhang, Wanli Zhu, Chenchao Ma, Kaixing Ai

**Affiliations:** ^1^Department of General Surgery, Shanghai Pulmonary Hospital, Tongji University School of Medicine, Shanghai, China; ^2^Department of Thoracic Surgery, Shanghai Pulmonary Hospital, Tongji University School of Medicine, Shanghai, China

**Keywords:** circ_0001666, circular RNA, circRNA, pancreatic cancer, microRNA-1251, EMT, SOX4

## Abstract

**Background:**

Pancreatic cancer (PC) is an aggressive malignancy and has a poor prognosis. Although emerging research has revealed that circular RNAs (circRNAs) are crucial modulators that control tumor development and metastasis, their functional involvement in PC has not been well characterized. Here, we examined whether and how circRNA circ_0001666 governs epithelial-mesenchymal transition (EMT) in PC.

**Methods:**

We investigated the effects of circ_0001666 on EMT and PC cell invasion by gain- and loss-of-function assays. We also explored the mechanisms underlying the functions of circ_0001666 in PC cells.

**Results:**

We found that circ_0001666 is highly expressed in PC tissues and PC cell lines. Patients with high circ_0001666 expression had shorter survival times. *In vitro* and *in vivo* experiments have demonstrated that upregulation of circ_0001666 facilitates PC cell proliferation, EMT and invasiveness, whereas knockdown of circ_0001666 exhibits opposite functions. Moreover, circ_0001666 is able to bind to miR-1251, thus increasing the expression of SOX4, which is a direct downstream effector of miR-1251. The oncogenic effects of circ_0001666 on EMT and PC cell invasion were rescued by miR-1251 overexpression.

**Conclusions:**

These results suggested that circ_0001666 acts as an oncogenic circRNA to promote EMT and invasion of PC cells through sponging miR-1251, and indicated that circ_0001666 could be explored as a potential therapeutic target for PC.

## Background

Pancreatic cancer (PC) is one of the most aggressive forms of cancer, and metastasis is the major cause of death (Garrido-Laguna and Hidalgo, 2015; Avula et al., 2020). To achieve a better treatment of PC, it is urgent to understand the mechanisms that drive tumorigenesis, progression and metastasis.

Epithelial-mesenchymal transition (EMT) refers to a process by which epithelial cells lose cell-cell adhesion, and gain migratory and invasive properties (Dongre and Weinberg, 2019). EMT plays a key role during the progression of epithelial cancers to metastatic cancers (Dongre and Weinberg, 2019). SOX4 is a member of the SOX (SRY-related HMG-box) family of transcription factors and increased SOX4 expression contributes to cellular transformation and EMT in many cancer types (Li et al., 2016; Liu et al., 2017; Zhang et al., 2020). SOX4 has a central role in promoting EMT, tumor growth and metastasis by directly regulating the expression of many genes involved in tumorigenesis, EMT, metastasis and cancer stemness (Hanieh et al., 2020). EZH2 is a catalytic subunit of the polycomb repressive complex 2, which catalyzes the trimethylation of histone3 lysine27 and mediates gene silencing (Chang and Hung, 2012). Inhibition of EZH2 attenuated EMT and metastasis in PC (Mody et al., 2016). It has been demonstrated that SOX4 functions directly as a transcriptional activator of the *EZH2* promoter and induces the expression of EZH2 in cancer cells (Tiwari et al., 2013).

MicroRNAs (MiRNAs) are endogenously expressed small non-coding RNAs that regulate gene expression by binding to the 3′-untranslated regions (3′-UTRs) of specific target messenger RNAs, resulting in mRNA degradation or inhibition of translation (Xu et al., 2019). MiRNAs have also been proved to modulate the EMT process in cancer cells (Zhang and Ma, 2012)12]. On the other hand, circular RNAs (circRNAs) are highly stable forms of non-coding RNAs that, unlike linear RNAs, form a covalently closed continuous loop (Dragomir and Calin, 2018). Emerging studies have shown that circRNAs function as miRNA sponges to trap miRNAs in human tumor cells (Dong P. et al., 2020). It has been reported that the expression of circ_0001666 was elevated in PC tissues compared with normal tissues (Song et al., 2020). To date, however, the functions and underlying mechanisms of dysregulated circ_0001666 expression in the EMT process of human PC remain unknown.

In this study, we discovered that the expression of circ_0001666 was significantly upregulated in PC tissues and positively correlated with worse patient prognosis. We demonstrated that circ_0001666 works as a sponge for miR-1251 to promote EMT and invasion of PC cells, at least in part via upregulating the levels of SOX4, a target of miR-1251. Our findings indicated that the circ_0001666/miR-1251/SOX4 signaling pathway was highly associated with the aggressive phenotypes of PC cells, thus providing a potential therapeutic strategy for treating PC.

## Materials and Methods

### PC Patient Specimens

A cohort of PC tissues and corresponding adjacent normal tissues were derived from 50 patients with PC who underwent surgery without preoperative chemotherapy or radiotherapy at Tongji University School of Medicine. All specimens were snap-frozen in liquid nitrogen upon collection. This study was approved by the Research Ethics Committee of Shanghai Pulmonary Hospital, Tongji University School of Medicine. Written informed consent was obtained from the patients before the study began.

### Cell Culture and Transient Transfection

The human PC cell lines (AsPC-1, PANC-1, SW-1990 and PaCa-2) and the immortalized human pancreatic duct epithelial cell line HPDE6-C7 were obtained from the American Type Culture Collection (Manassas, VA, United States). All cell lines were maintained in RPMI1640 medium (Invitrogen, Carlsbad, CA, United States) supplemented with 10% fetal bovine serum (FBS, Invitrogen). These cells were kept in a 37°C incubator with 5% CO2.

Cells were transfected with Lipofectamine 3,000 reagent (Invitrogen). The vectors overexpressing circ_0001666 (or SOX4), and the control vector were purchased from Genepharma (Shanghai, China). SiRNAs against circ_0001666 (or SOX4), the miR-1251 mimic and miR-1251 inhibitor were purchased from Ribobio (Guangzhou, China).

### RNA Extraction and Real-Time PCR (qRT-PCR)

Total RNA from cells and freshly frozen tissues was isolated using TRIzol reagent (Invitrogen). The total RNA was reverse transcribed using the PrimeScript RT Master Mix (Takara, Dalian, China). Total RNA was incubated at 37°C with 3 U/μg RNase R (Geneseed, Guangzhou, China) for 30 min. Nuclear and cytoplasmic RNA fractionation was isolated using NE-PER Nuclear and Cytoplasmic Extraction Reagents (Thermo Fisher Scientific, CA. United States). The expression of circ_0001666 and SOX4 was quantified using SYBR Pre-mix Ex Taq quantitative PCR kit (Takara, Dalian, China). *GAPDH* was used as an internal control for circ_0001666 and SOX4. The expression of miR-1251 was determined using the mirVanaTM qRT-PCR microRNA Detection Kit (Ambion, Austin, TX, United States) and normalized to U6. The primer sequences were obtained from Ribobio (Guangzhou, China).

### Western Blotting Analysis

PC cells were lysed with a RIPA buffer (Beyotime, Beijing, China). Equal amounts (30 μg) of protein were separated on 15% SDS-PAGE gels, and transferred onto PVDF membranes (Millipore, Bedford, MA, United States). The membranes were incubated with the primary antibodies, including anti-E-cadherin (Cell Signaling, MA, United States), anti-Vimentin (Cell Signaling), anti-EZH2 (Cell Signaling), anti-SOX4 (Cell Signaling), and anti-β-actin (Cell Signaling) at 4°C overnight. Afterward, the secondary antibodies were added and blot signals were visualized with an ECL detection system (Amersham Biosciences, Buckinghamshire, United Kingdom).

### Cell Proliferation Assay

Cell proliferation was detected by Cell Counting Kit-8 assay (CCK-8, Dojindo, Kumamoto, Japan). The cells were plated in 96-well plates. After 96-h incubation, 10 μl CCK-8 solutions were added and the absorbance at 450 nm was determined using a microplate reader (Tecan Austria GmbH 5082, Grodig, Austria).

### Invasion Assay

For the transwell invasion assay, PC cells suspended in serum-free medium were seeded in the upper chambers (8-μm pore size, Corning Costar Co, CA, United States). 750 μl medium containing 10% FBS was placed in the lower chamber, as previously reported (Dong et al., 2018). After incubation for 24 h, the cells were fixed, stained and counted using an inverted microscope.

### Animal Experiment

All animal experiments were approved by the Institutional Review Board of Shanghai Pulmonary Hospital, Tongji University School of Medicine. Four-week-old nude mice were purchased from Beijing HFK Bioscience (Beijing, China), and randomly divided into two groups (*n* = 5). PC cells transfected with circ_0001666 plasmid (or control vector), or with circ_0001666 siRNA (or control siRNA), were subcutaneously injected into the right flank of nude mice, respectively. The volume of the tumors was measured every week after implantation. The tumor volume was calculated using the following formula: tumor volume [mm^3^] = (length [mm]) × (width [mm])^2^ × 0.5. The mice were sacrificed after 21 days. The tumors were removed, and the tumor weight was measured.

### Dual-Luciferase Reporter Assay

The luciferase reporter plasmids containing wild-type (WT) circ_0001666 fragment, mutant (MUT) circ_0001666 fragment with a mutated miR-1251 binding site, WT *SOX4* 3**′-**untranslated region (3**′-**UTR) fragment, or MUT *SOX4* 3**′-**UTR fragment with a mutated miR-505 binding site, were purchased from Ribobio (Guangzhou, China). PC cells were co-transfected with the reporter plasmids with miR-1251 mimic, miR-1251 inhibitor or the respective negative controls, along with the Renilla luciferase plasmid pRL-CMV (Promega, WI, United States), using the Lipofectamine 3,000 (Invitrogen). The luciferase activities were measured using the Dual-Luciferase Reporter Assay System (Promega). Firefly luciferase activities were normalized to Renilla luciferase activities.

### RNA Immunoprecipitation Assay

RNA Immunoprecipitation (RIP) assay was conducted with Magna RIP RNA-Binding Protein Immunoprecipitation Kit (Millipore, MA, United States). PC cells were lysed in a RIP-lysis buffer. Then, the magnetic beads conjugated with an anti-Ago2 antibody (Millipore) or a control anti-IgG antibody (Millipore) were added to cell lysates, and the lysates were rotated overnight. After incubating with proteinase K for 30 min, RNAs were purified and the levels of circ_0001666 and miR-1251 were quantified using qRT-PCR analysis.

### Statistical Analysis

All results were representative results from at least three independent experiments. Data are expressed as the mean ± standard deviation. Significant differences for the mean values between groups were determined using Student’s *t*-tests and one-way ANOVA. Data analysis was performed by using SPSS 25.0 software. *P* < 0.05 was considered statistically significant.

## Results

### Upregulation of circ_0001666 in PC Tissues and PC Cells

**FIGURE 1:**
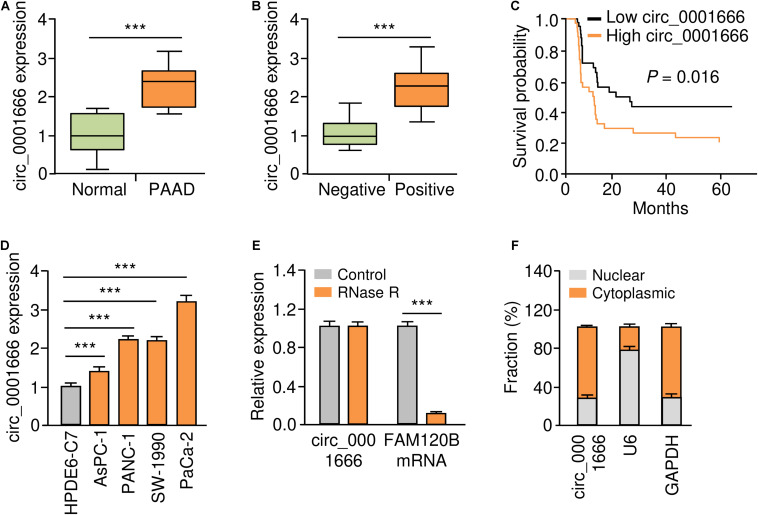
Upregulation of circ_0001666 in PC tissues and PC cells. **(A)** The expression of circ_0001666 between PC tissues and adjacent normal tissues were assessed using qRT-PCR analysis. **(B)** qRT-PCR analysis of circ_0001666 expression in PC tissues from patients with or without lymph node metastasis. **(C)** Kaplan-Meier curves showed that higher expression of circ_0001666 was associated with poor overall survival in PC patients. **(D)** qRT-PCR assay of circ_0001666 expression in multiple PC cell lines and a normal pancreatic cell line HPDE6-C7. **(E)** qRT-PCR analysis of circ_0001666 and FAM120B expression in PaCa-2 cells in the presence or absence of RNase treatment. **(F)** Cytoplasmic and nuclear fractionation assay showed that circ_0001666 was mainly located in the cytoplasm. *P < 0.05, **P < 0.01, ***P < 0.001.

According to the annotation of circBase database^1^, we found that *FAM120B* is the host gene of circ_0001666. Circ_0001666 is located at chr6:170626457-170639638, and the length of its mature transcript is 2038 nt. In order to understand the role of circ_0001666 in PC, we used qRT-PCR assay to examine the expression of circ_0001666 in 50 pairs of primary PC tissues and adjacent non-cancerous tissues derived from PC patients. We found that circ_0001666 was significantly overexpressed in PC samples compared with adjacent normal tissues (Figure 1A). We next evaluated the association between circ_0001666 expression and clinical-pathological parameters, and found that higher expression of circ_0001666 was positively correlated with the presence of lymph node metastasis (Figure 1B). Survival analysis revealed that those patients with high (above the median) circ_0001666 levels displayed lower overall survival rates (Figure 1C).

The level of circ_0001666 in four PC cell lines and one normal pancreatic duct epithelial cell line HPDE6-C7 was further confirmed by qRT-PCR assay. As expected, circ_0001666 were consistently upregulated in PC cell lines compared with HPDE6-C7 cells (Figure 1D). As shown in Figure 1E, circ_0001666 resisted the digestion of RNase R, while the linear form of *FAM120B* mRNA was digested by RNase sharply. We detected the location of circ_0001666 in PC cells, and found that this circRNA was mainly localized in the cytoplasm (Figure 1F). These results show that circ_0001666 is overexpressed in PC tissues and cell lines, and high circ_0001666 expression is correlated with poor prognosis in PC patients.

### Knockdown of circ_0001666 Inhibits the Proliferation of PC Cells

**FIGURE 2:**
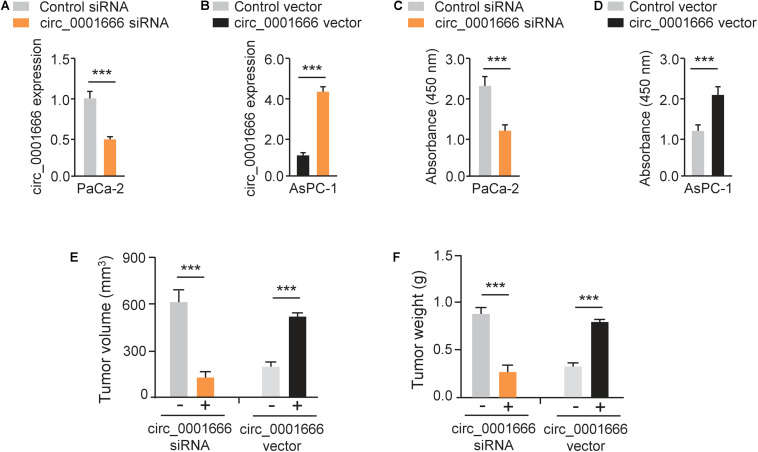
Knockdown of circ_0001666 inhibits the proliferation of PC cells. **(A)** qRT-PCR assay of circ_0001666 expression in PaCa-2 cells transfected with circ_0001666 siRNA or control siRNA. **(B)** qRT-PCR assay of circ_0001666 expression in AsPC-1 cells transfected with circ_0001666 vector or control vector. **(C,D)** CCK-8 assay in PaCa-2 **(C)** or AsPC-1 **(D)** cells transfected as indicated. **(E,F)** The tumor volume **(E)** and tumor weight **(F)** of the tumors derived from PaCa-2 or AsPC-1 cells. ***P < 0.001.

We characterized the role of circ_0001666 in PC cells. Circ_0001666 knockdown and overexpression models were constructed in PaCa-2 (Figure 2A) cells and AsPC-1 cells (Figure 2B), respectively. The knockdown or overexpression efficiency was validated by qRT-PCR assay (Figure 2B). Then, the cell proliferation rate in these cells was tested using CCK-8 assay. Knockdown of circ_0001666 significantly reduced the proliferation of PaCa-2 cells (Figure 2C), and overexpression of circ_0001666 significantly improved the proliferation of AsPC-1 cells (Figure 2D). In addition, to evaluate the role of circ_0001666 in tumor growth *in vivo*, nude mice were injected with PaCa-2 cells that were transfected with circ_0001666 siRNA or control siRNA, and with AsPC-1 cells that were transfected with circ_0001666 overexpression vector or the control vector. The volume (Figure 2E) and weight (Figure 2F) of tumors were markedly lower in circ_0001666 knockdown group than the control group, while the volume (Figure 2E) and weight (Figure 2F) of tumors were significantly higher in circ_0001666 overexpression group than the control group. Our results suggest that knockdown of circ_0001666 inhibits PC growth both *in vitro* and *in vivo*.

### Knockdown of circ_0001666 Represses EMT in PC

**FIGURE 3:**
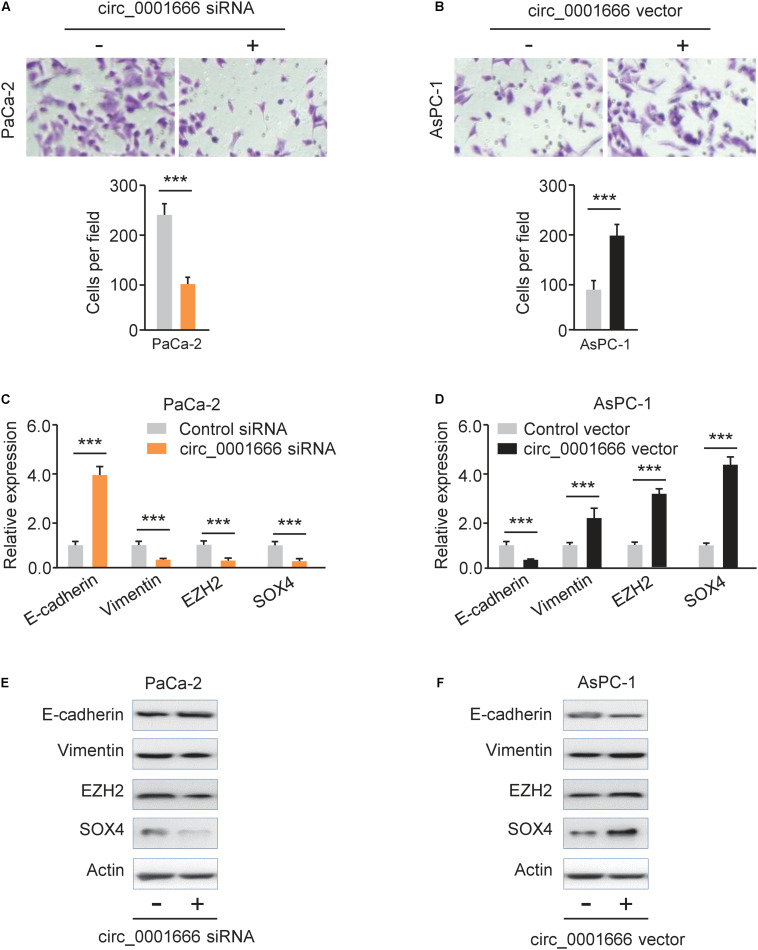
Knockdown of circ_0001666 represses EMT in PC cells. **(A)** The invasive abilities of PaCa-2 cells following knockdown of circ_0001666 were evaluated using cell invasion assay. **(B)** The invasive abilities of AsPC-1 cells following overexpression of circ_0001666 were evaluated using cell invasion assay. **(C,D)** qRT-PCR analysis of EMT-related genes in PaCa-2 cells following knockdown of circ_0001666 **(C)**, and in AsPC-1cells following overexpression of circ_0001666 **(D)**. **(E,F)** Western blotting analysis of EMT-related genes in PaCa-2 cells following knockdown of circ_0001666 **(E)**, and in AsPC-1cells following overexpression of circ_0001666 **(F)**. ***P < 0.001.

EMT is one of the major mechanisms for the enhanced invasion ability of cancer cells (Dongre and Weinberg, 2019). To evaluate the potential effects of circ_0001666 on EMT, we performed a cell invasion assay and demonstrated that knockdown of circ_0001666 significantly inhibited cell invasion in PaCa-2 cells (Figure 3A). However, the invasive abilities of AsPC-1 cells were significantly enhanced following overexpression of circ_0001666 (Figure 3B). Furthermore, the mRNA expression of EMT markers in circ_0001666-silenced PaCa-2 cells or circ_0001666-overexpressing AsPC-1 cells was investigated using qRT-PCR assay. The expression of E-cadherin was increased, while the expression of Vimentin was decreased in circ_0001666-silenced PaCa-2 cells (Figure 3C). Consistently, overexpression of circ_0001666 led to the downregulation of E-cadherin and upregulation of Vimentin in AsPC-1 cells (Figure 3D). These results were verified using western blotting analysis (Figures 3E,F). Our data suggest the role of circ_0001666 in promoting EMT in PC cells.

### Circ_0001666 Acts as a miRNA Sponge of miR-1251 to Promote PC Cell Invasion

**FIGURE 4:**
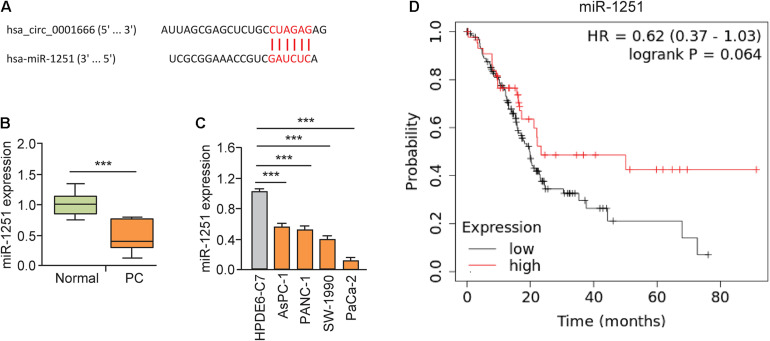
MiR-1251 is downregulated in PC tissues and associated with a better prognosis. **(A)** The predicted miR-1251 binding site in the circ_0001666 sequence. **(B)** The expression of miR-1251 in PC tissues and adjacent normal tissues, as measured by qRT-PCR assay. **(C)** qRT-PCR analysis of miR-1251 expression in PC cell lines and normal pancreatic cells. **(D)** The prognostic role of miR-1251 expression in PC was explored using the KM plotter database. ***P < 0.001.

Here, we explored whether circ_0001666 promotes PC progression by sponging miRNAs. First, we predicted the potential miRNAs that might bind to circ_0001666 using the CircInteractome database. Circ_0001666 had a complementary binding sequence to miR-1251 (Figure 4A). The expression of miR-1251 in PC tissues was lower than that in the adjacent normal tissues (Figure 4B). Meanwhile, reduced expression of miR-1251 was observed in multiple PC cell lines compared with HPDE6-C7 cells (Figure 4C). The survival curves from Kaplan-Meier Plotter database showed that lower levels of miR-1251 predicted a poorer outcome in PC patients (Figure 4D).

**FIGURE 5:**
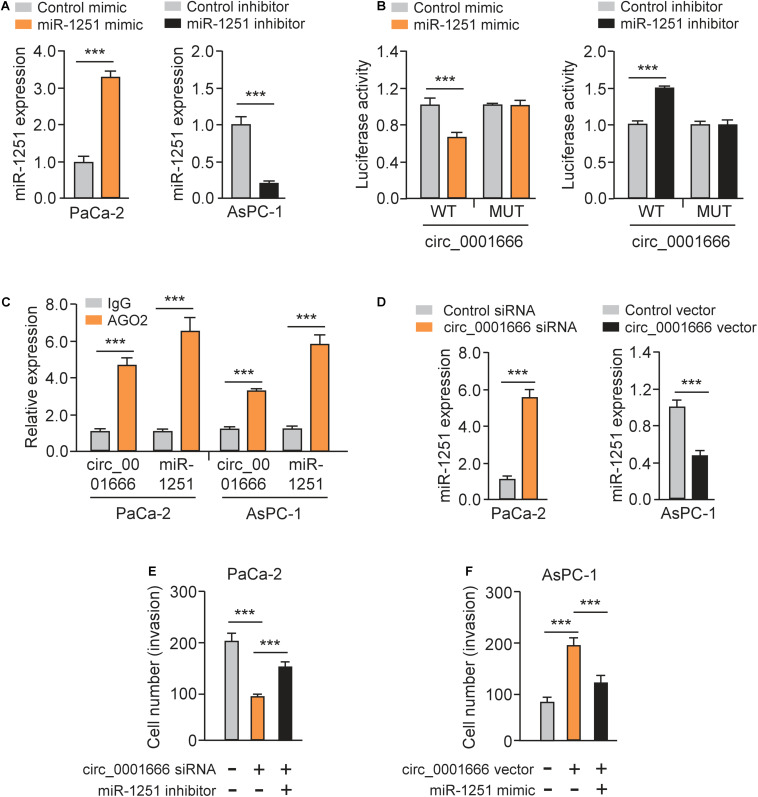
Circ_0001666 acts as a miRNA sponge of miR-1251 to promote PC cell invasion. **(A)** The expression of miR-1251 in PC cells after overexpression or knockdown of miR-1251. **(B)** The effects of miR-1251 mimic, miR-1251 inhibitor, or the negative control on the luciferase activity of the WT or MUT circ_0001666 sequence were detected in PC cells using the luciferase reporter assay. **(C)** RIP assay was performed in PC cells, followed by qRT-PCR assay. **(D)** The expression of miR-1251 in PC cells after knockdown or overexpression of circ_0001666. **(E,F)** Invasion abilities of PaCa-2 cells transfected with or without circ_0001666 siRNA, with (or without) miR-1251 inhibitor **(E)**, or in AsPC-1 cells transfected with (or without) circ_0001666 vector, with (or without) miR-1251 mimic **(F)**. ***P < 0.001.

The luciferase reporter assays illustrated that overexpression of miR-1251 suppressed the luciferase activity of WT circ_0001666 sequence, and knockdown of miR-1251 increased the luciferase activity of WT circ_0001666 sequence (Figures 5A,B). However, modulation of miR-1251 expression did not change the luciferase activity of mutated circ_0001666 sequence (Figures 5A,B), suggesting a direct interaction between circ_0001666 and miR-1251 in PC cells. Subsequent RIP assay was used to pull down circ_0001666 and miR-1251 with an anti-AGO2 antibody (IgG as a negative control) in PaCa-2 and AsPC-1 cells. We found that both circ_0001666 and miR-1251 were efficiently pulled down by anti-AGO2 antibody compared with anti-IgG (Figure 5C). These results demonstrated that circ_0001666 binds to miR-1251 in PC cells.

Furthermore, knockdown of circ_0001666 upregulated the expression of miR-1251, while overexpression of circ_0001666 displayed an opposite effect (Figure 5D). Importantly, co-transfection of miR-1251 inhibitor could rescue cell invasion that was suppressed by circ_0001666 knockdown (Figure 5E). Additionally, overexpression of circ_0001666 led to increased cell invasion, but this change was reversed by ectopic expression of miR-1251 (Figure 5F). Taken together, our results suggest that circ_0001666 acts as a sponge of miR-1251 to promote PC cell invasion.

### MiR-1251 Regulates EMT and PC Cell Invasion by Targeting SOX4

**FIGURE 6:**
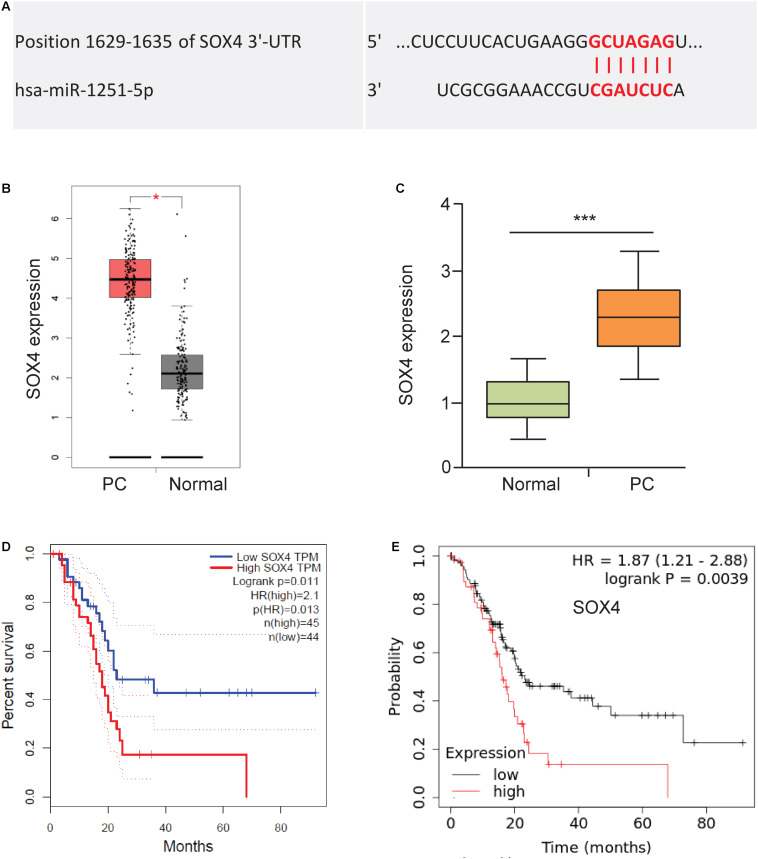
Upregulation of SOX4 is correlated with worse patient survival. **(A)** Predicted binding site between miR-1251 and SOX4 (TargetScan database). **(B)** The expression of SOX4 expression in PC tissues and adjacent normal tissues (GEPIA database). **(C)** SOX4 mRNA expression in PC tissues and adjacent normal tissues was tested using qRT-PCR assay. **(D,E)** Kaplan-Meier curves showed the overall survival of PC patients expressing higher or lower SOX4 expression (**D**: GEPIA database; **E**: KM Plotter database). ***P < 0.001.

To explore the functional downstream target of miR-1251, we predicted the possible binding sites using the TargetScan database. We found that SOX4 might be a target of miR-1251 (Figure 6A). Using the GEPIA database, we explored SOX4 mRNA expression between PC and normal tissues. The results demonstrated that the expression of SOX4 was significantly higher in PC tissues (Figure 6B). We confirmed the overexpression of S100A in PC tissues using qRT-PCR assay (Figure 6C). We next used GEPIA and Kaplan-Meier plotter databases to analyze the prognostic values of the mRNA expression of SOX4 in PC patients. Higher mRNA expression of SOX4 was significantly associated with shorter overall survival of PC patients (Figures 6D,E).

**FIGURE 7:**
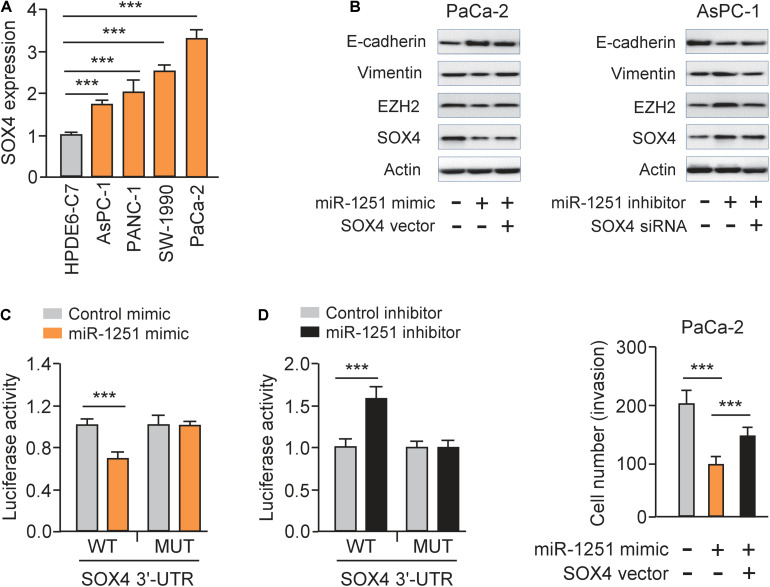
MiR-1251 regulates EMT and PC cell invasion by targeting SOX4. **(A)** qRT-PCR assay of SOX4 expression in PC cell lines and a normal pancreatic cell line HPDE6-C7. **(B)** Western blotting analysis of E-cadherin, Vimentin, EZH2, SOX4, and β-actin expression in PC cells transfected as indicated. **(C)** Luciferase reporter assay for luciferase activities of the WT or MUT SOX4 3′-UTR reporter in PC cells transfected as indicated. **(D)** Cell invasion assay in PC cells transfected as indicated. ***P < 0.001.

As expected, we observed a significantly high expression of SOX4 in PC cell lines compared to normal cells (Figure 7A). Since SOX4 regulates EMT of cancer cells by controlling EZH2 expression (Hanieh et al., 2020), we investigated whether miR-1251 could modulate the protein expression of EMT-related markers, SOX4 and EZH2 in PC cells. Our western blotting assay showed that overexpression of miR-1251 significantly enhanced the levels of E-cadherin, but downregulated the expression of Vimentin, SOX4 and EZH2 (Figure 7B). Knockdown of miR-1251 had opposite effects (Figure 7B). Moreover, our rescue experiments demonstrated that the ability of miR-1251 in regulating the expression of these proteins was largely abrogated by overexpression of SOX4, and the impact of miR-1215 inhibition on gene expression was reversed by transfection with SOX4 siRNA (Figure 7B).

Luciferase reporter assays were performed and the results showed that transfection with miR-1251 mimic significantly decreased the luciferase activity of the WT SOX4 3′-UTR, while the introduction of miR-1251 inhibitor could remarkably increase it, but not that of the MUT SOX4 3′-UTR in PC cells (Figure 7C). These data clearly suggested that miR-1251 suppresses SOX4 expression in PC cells by directly binding to 3′-UTR of SOX4 mRNA. Subsequent rescued experiments have shown that overexpression of SOX4 significantly reversed the ability of miR-1251 to suppress the invasion of PaCa-2 cells (Figure 7D). In line with the role of circ_0001666 as an upstream regulator of miR-1251, our western blotting experiments revealed that overexpression of circ_0001666 resulted in the upregulation of SOX4 and EZH2 in AsPC-1 cells (Figure 3E). However, silencing of circ_0001666 downregulated the protein expression of SOX4 and EZH2 (Figure 3E). These results support the notion that circ_0001666 increased the expression of SOX4 and EZH2 by downregulating miR-1251 expression.

**FIGURE 8:**
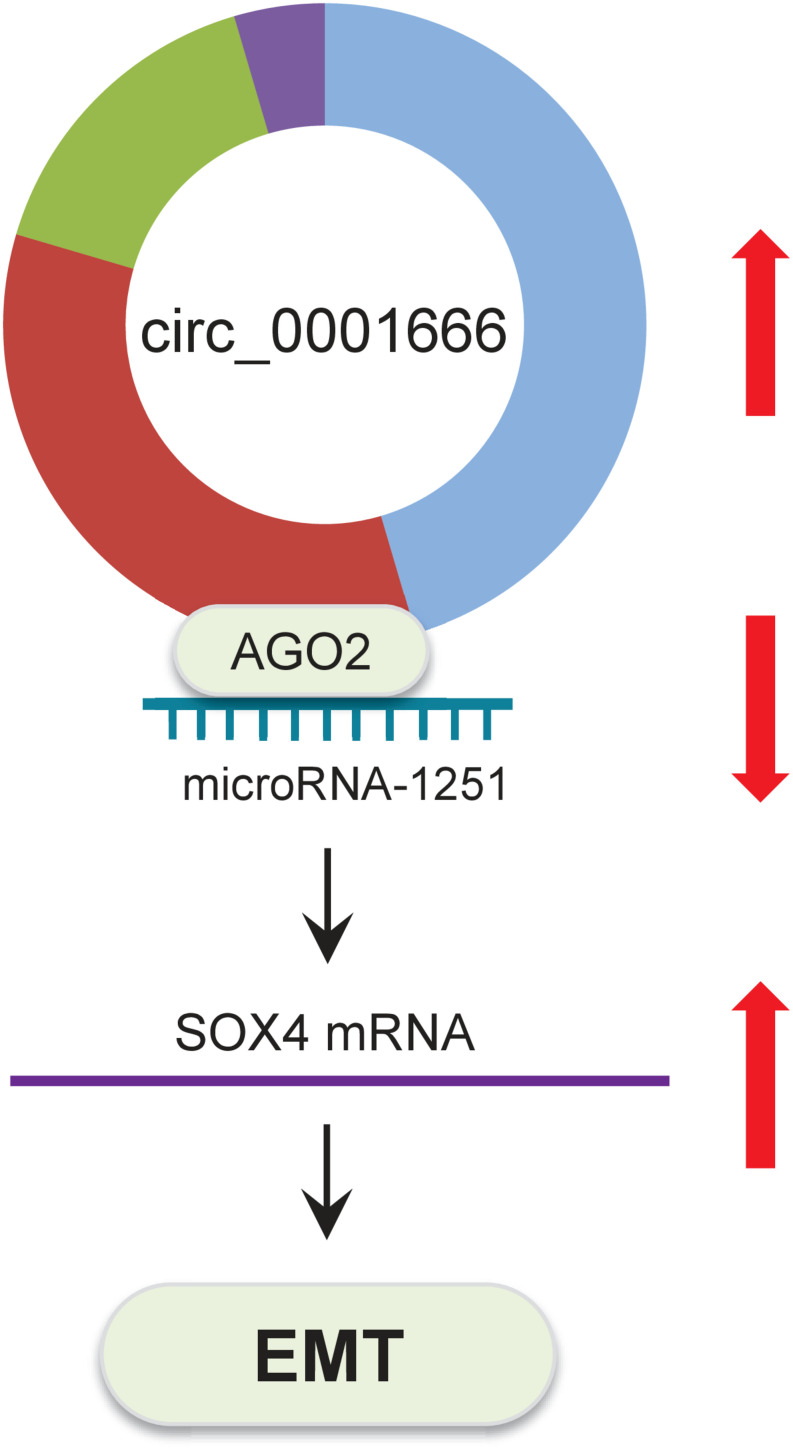
Schematic representation of the proposed mechanism by which circ_0001666 mediates EMT in PC cells. Circ_0001666 acts as a miR-1251 sponge to upregulate the expression of SOX4, thereby promoting EMT and PC cell invasion.

In summary, all of these results indicated that circ_0001666 promotes EMT and invasive properties of PC cells through sponging miR-1251 and enhancing SOX4 expression (Figure 8).

## Discussion

Recently, larger numbers of circRNAs have been discovered by RNA sequencing, and many of the differentially expressed circRNAs were shown to have oncogenic or tumor suppressor functions *in vitro* and *in vivo* (Dong P. et al., 2020). Altered expression of circRNA is closely related to oncogenesis, progression and metastasis in PC (Rong et al., 2021). Although the upregulation of circ_0001666 has already been recognized in PC tissues (Song et al., 2020), its functional relevance and molecular mechanisms remain undetermined. In this study, we verified the overexpression of circ_0001666 in PC samples and found that high circ_0001666 expression was associated with poor prognosis in PC patients. The small sample size was a limitation of our study. Our future research based on large sample sizes should be performed to evaluate the clinical significance of circ_0001666 in PC patients. Furthermore, we showed that circ_0001666 works as a sponge for miR-1251 to induce EMT and invasion of PC cells, at least in part via upregulating the levels of SOX4, a target of miR-1251. Our results suggest that circ_0001666 is acting as an oncogenic circRNA through upregulation of SOX4 by inhibiting miR-1251 expression.

Most studies have validated that circRNAs are functional transcripts that could regulate gene expression through modulating gene transcription and splicing, titrating miRNAs, interacting with proteins, and acting as templates for the synthesis of polypeptides (Chen, 2020). Many circRNAs accumulate in the cytoplasm and function as competing endogenous RNAs, which can bind and sponge target miRNAs to prevent these miRNAs from binding and suppressing their target mRNAs (Chen, 2020). For example, elevated expression of circ_0066147 enhanced the proliferation, invasion, migration, and EMT process of PC cells *in vitro* by sponging miR-330-5p (Xu et al., 2020). Circ_0006948 is a sponge of miR-490 in esophageal squamous cell carcinoma cells, and it upregulates the expression of oncogene HMGA2 to induce EMT by sequestering miR-490 (Pan et al., 2019). These findings are in accordance with our observations that, in PC cells, circ_0001666 functions as a competing endogenous RNA to decrease miR-1251 expression, thus promoting SOX4-dependent EMT. On the other hand, the function of circRNA as protein scaffold or sequester protein has been exemplified by numerous examples (Chen, 2020). CircPABPN1 is reported to sequester HuR, thereby serving as a decoy for HuR and impairing PABPN1 translation in cervical cancer cells (Abdelmohsen et al., 2017). HuR recognizes and binds to the AU-rich elements in its target mRNAs, and enhances mRNA stability and translation (Mukherjee et al., 2011). HuR binds to the 3′-UTR of the mRNA of the EMT inducer Snail, resulting in stabilization of *Snail* mRNA and enhanced Snail protein expression and thus promoting EMT, metastasis, and formation of stem-like PC cells (Dong R. et al., 2020). Using the CircInteractome database, we have found that mature circ_0001666 hosts multiple binding sites for several RNA-binding proteins like HuR (data not shown). The association between circ_0001666 and HuR (or other proteins) in PC cells represents an interesting topic for future research.

The published studies showed that miR-1251 significantly affected the prognosis of PC patients and high expression of miR-1251 is associated with favorable survival (Jia et al., 2020). In line with this data, we determined that miR-1251 impairs the EMT features of PC cells. Of note, miR-1251 was also reported to promote tumor growth and metastasis of hepatocellular carcinoma (Han et al., 2020), and facilitate carcinogenesis and autophagy in ovarian cancer (Shao et al., 2019). These data imply that that miR-1251 has complex roles in regulating tumor initiation and progression depending on cell type, and further characterization of its target genes would provide insights in miR-1251-reduced EMT in PC cells.

The *SOX4* gene is frequently amplified and overexpressed in more than 20 types of malignancies, where SOX4 acts as an oncogene (Moreno, 2020). SOX4 interacts with multiple other transcription factors, rendering its impacts on gene expression and leading to the promotion of cell survival, cancer stemness, EMT and metastasis (Moreno, 2020). Importantly, knockdown of SOX4 with siRNA in PC cells resulted in restricted tumor growth both *in vitro* and in mice (Huang et al., 2012). In addition, miR-129 and miR-335 target *SOX4* mRNA for degradation in PC cells (Huang et al., 2012). Our study added new evidence to support the oncogenic role of SOX4 in PC cells, and its overexpression is partially due to the repression of miR-1251. Thus, SOX4 might globally regulate the expression of many genes (such as EZH2) (Hanieh et al., 2020; Moreno, 2020), thereby contributing to EMT and PC metastasis.

In conclusion, we described a novel circ_0001666/miR-1251/SOX4 regulatory pathway in PC cells. Circ_0001666 represses miR-1251 expression to increase SOX4 expression, subsequently inducing EMT and enhancing PC cell invasion (Figure 8). Overall, the components of this pathway could be promising targets for developing new PC therapies.

## Data Availability Statement

The original contributions presented in the study are included in the article/supplementary material, further inquiries can be directed to the corresponding author/s.

## Ethics Statement

The studies involving human participants were reviewed and approved by the Research Ethics Committee of Tongji University School of Medicine. The patients/participants provided their written informed consent to participate in this study. The animal study was reviewed and approved by the Institutional Review Board of Tongji University School of Medicine.

## Author Contributions

KA designed the experiments. RZ performed the experiments. WZ and CM analyzed the data. All authors read and approved the final manuscript.

## Conflict of Interest

The authors declare that the research was conducted in the absence of any commercial or financial relationships that could be construed as a potential conflict of interest.

## Footnotes

1 http://www.circbase.org/
